# Predictors of weight loss after an intensive lifestyle intervention program in obese patients: a 1-year prospective cohort study

**DOI:** 10.1186/1477-7525-11-165

**Published:** 2013-10-03

**Authors:** Tor-Ivar Karlsen, Maryon Søhagen, Jøran Hjelmesæth

**Affiliations:** 1 Department of Health and Nursing Sciences, University of Agder, Jon Lilletuns vei 9, Grimstad, 4879, Norway; 2 Department of Psychosocial Health, University of Agder, Grimstad, Norway; 3 Department of Public Health, Sport and Nutrition, University of Agder, Kristiansand, Norway; 4 Morbid Obesity Centre, Vestfold Hospital Trust, Tønsberg, Norway

**Keywords:** Morbid obesity, Predictors, Lifestyle intervention, Weight loss, Health related quality of life

## Abstract

**Background:**

Studies of lifestyle intervention programs in morbid obesity report large variations in weight loss outcomes. This is reported not only between but also within standardized programs. Such reports point to participants’ characteristics as possible predictors of this outcome. The aim of this prospective cohort study was to identify predictors of weight loss after a 1-year partly residential intensive lifestyle intervention program (ILI).

**Methods:**

Morbidly obese patients (n=199), all Caucasian, 71% women, mean (SD) age 45.2 (11.1) years, body mass index (BMI) 42.0 (6.2) kg/m^2^, and excess body weight (>BMI=25 kg/m^2^) 49.4 (19.6) kg, were referred from public hospitals to a rehabilitation center and enrolled consecutively. The 1-year ILI comprised of four (n=104) or five (n=95) stays at the rehabilitation center. In both cases there was one main stay for 4 weeks and the remaining stays lasted 1 week each. In the home periods the patients were followed up by telephone and by their general practitioners (GP). The patients were also encouraged to use a predefined paper based diary. Health related quality of life (HRQL), diagnostic, anthropometric, socio-demographic, psychosocial and intervention characteristics were measured at baseline, 12 weeks and 1 year. Multiple linear regression analyses were performed to extract possible predictors of weight loss at 1-year. Direct and indirect effects of these predictors were tested through structural equation modeling.

**Results:**

The mean (SD) 1-year weight loss was 10 (11) kg, corresponding to an 8 (8) % reduction of body weight from baseline. Mean excess weight loss (EWL) was 20 (22) % ranging from 104% to -77%. The adherence to a diary (r=.16), type 2 diabetes (r=-.14) and frequency of GP-visits (r=.23) were significantly associated with EWL at 12 weeks. Predictors of 1-year EWL were 12 week EWL (r=.66), occupational status (r=.11), age (r=.19), and mental HRQL (r=-.16), all p<.05. The path model explained 50% of the variation (r^2^=.50) of 1-year EWL.

**Conclusion:**

Larger 12 week weight loss, being employed, lower mental HRQL and being older predicts larger weight loss after 1 year in morbidly obese patients following ILI. Not having type 2 diabetes, using a diary combined with regular GP follow-up influence the 12-week weight loss.

**Trial registration:**

Clinicaltrials.gov: NCT00477399

## Introduction

Obesity is an excessive accumulation of fat with body mass index (BMI) ≥ 30 kg/m^2^ as a threshold [1]. Morbid obesity is understood as a BMI ≥ 35 kg/m^2^ with at least one comorbid condition or BMI ≥ 40 kg/m^2^[2]. The prevalence of obesity and morbid obesity is increasing in most countries, and globally around 500 million people are obese [3]. Although bariatric surgery has been shown to be more effective than lifestyle intervention at improving weight loss and reducing comorbidities [4,5] not all morbidly obese subjects can be treated surgically.

Even though a modest weight loss of 5-10% of the baseline weight is reported to have significant positive effects on comorbid conditions [6], many patients undergoing lifestyle interventions do not reach this goal [7]. Lifestyle intervention is comprised of a diversity of approaches, from simple diets to comprehensive psychosocially oriented methods, and from internet based to intensive residential treatment programs. These different weight loss programs report large variations of achieved weight loss, but also large variations between participants in the same programs [7]. This emphasizes participants’ characteristics as possible predictors of weight loss. A verification of such predictors may help clinicians to identify patients at risk of not reaching treatment goals and enable them to tailor a lifestyle program to meet their patients’ needs.

The number of potential predictors is large [8]. Co-morbid conditions, like arthrosis, diabetes, depression, anxiety and sleep apnea can, theoretically, predict lower weight losses due to the deterioration of the individual’s physiological, psychological or social abilities. Likewise, an individuals’ obesity history may limit their physical and social activity. Age may also be an important factor in successful weight loss, in addition to socioeconomic factors like employment, income, education and social status. An individual’s quality of life may affect the outcome of a weight loss program through loss of motivation. Likewise, psychosocial factors may have an impact on the mental strain changing lifestyle can bring about.

The majority of studies on predictors of weight loss have focused on bariatric surgery [9]. However, a review of psychosocial pre-treatment predictors of weight control [7] included 29 studies with various predictor groups; eating patterns, motivation, outcome expectancies, locus of control, body image and self-esteem, psychological health and perceived stress, social support, quality of life, and physical activity. The review found consistent evidence that the number of previous dieting and weight loss attempts, together with self-motivation, general efficacy and autonomy, were predictors of weight control. Notably, eating patterns, depression and mood disturbances, social support, and personality styles did not predict weight outcomes. The reviewers recommended that future research apply a bio-psycho-social model and that the various predictors should be analyzed in a more sophisticated way allowing moderator variables to be uncovered.

Such recommendations are constructive. In a real life context, as in a weight loss program, individual physiological and psychological factors, often genetically influenced, interact with social and environmental factors, giving a multitude of individual responses to both the magnitude and rate of weight changes. There is no evidence in the research literature of a single variable strongly predicting weight loss; variables interact and models have to be developed in order to adjust for and incorporate the interdependencies among the variables.

The aim of this study was to develop a conceptual model of predictors of weight loss after a 1-year psychosocially oriented partly residential intensive lifestyle intervention program (ILI) for morbid obesity.

## Material and methods

### Study design and participants

The study was a 1-year prospective cohort study of 200 morbidly obese patients referred to a Norwegian rehabilitation center (Evjeklinikken AS) from internists at public hospitals. The inclusion criterion was a condition of morbid obesity, and participants were recruited consecutively from May 2006 to November 2010. All patients were Caucasian, with all but one patient completing the program, leaving n=199 eligible for analysis.

The study (clinicaltrials.gov identifier NCT00477399) was conducted after written informed consent was obtained from all the participants according to the Helsinki protocol. The study was approved by the Norwegian Regional Committee for Medical and Health Research Ethics (S-05175).The cohort is to be followed for 4 more years.

### Intervention

The overall goal of the ILI was to attain a weight loss of ≥ 10% of baseline patient weight. The intervention aimed to encourage patients to increase their physical activity and to reduce or normalize their eating habits. The intention of the program was to empower individuals towards changing lifestyle by increasing self-efficacy and by improving their self-esteem so that they could better deal with their weight problem.

**Figure 1:**
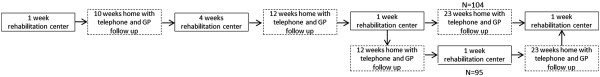
Schedule of stays during the 1-year intensive lifestyle intervention program at the rehabilitation center.

The 1-year ILI comprised a combination of stays at a rehabilitation center, telephone follow up and GP-consultations while patients stayed home. Due to structural changes of the ILI throughout the study period the first 104 patients underwent 4 stays and 95 patients underwent 5 stays, one main stay for four weeks and the remaining stays lasting one week (Figure 1). The daily schedule was divided between organized physical activity (3–4 hours) varying in intensity from light, moderate to vigorous, and various psychosocially oriented interventions with a motivational approach. The intervention included individual consultations with a medical doctor, registered dieticians, physiotherapists, and mental-health nurses, all trained in motivational interviewing. The intervention also included group sessions focusing on nutrition, physical activities, comorbidities, and emotional feelings towards obesity and their lifestyle. No special diet or weight loss drugs were prescribed, but patients were encouraged to reduce their daily total energy intake and follow the nutrition guidelines from the Norwegian National Council of Nutrition [9], which recommend that the daily intake of protein, fat, carbohydrate and alcohol should account for 10–20, > 30, 50–60 and < 5% of energy consumed, respectively. Nutritional advices were conveyed to the patients through individual consultations and group sessions with Registered Dieticians*.* Outside their stays at the rehabilitation center patients where contacted by phone once every second week and were also encouraged to consult their general practitioner for weight measurement and follow-up every four weeks. They were also encouraged to self-monitor eating habits and physical activities on a daily basis in a paper based diary. In the diary patients were to mark what kind of meals they had eaten, approximately how many steps they had taken, main activities and “food temptations”. They were also encouraged to write a few words describing their emotions that day. The patients were also instructed to bring the diary to their GP for monthly signing and follow up.

### Variables, sources and measurement

Data from patient medical records was used to assess each participant’s baseline sociodemographic and socioeconomic status, anthropometric characteristics, age at onset of obesity, maximum weight in kg, age of maximum weight, 12 weeks process variables (weight loss, adherence to diary and frequency of GP-visits) and weight at 1 year.

Weight and body fat were measured by “Tanita Body Composition Analyzer TBF-310”. Weight was measured in the morning before breakfast, after urinating, and after an easy walk of approximately 20 min. Referral documentation from hospital internists gave detailed information on various comorbidities. The diagnoses were categorized in accordance with the ICD-10 codes.

Obesity specific health related quality of life (HRQL) was assessed by the Obesity and Weight Loss Quality Of Life (OWLQOL) questionnaire [10], which primarily assesses emotions and feelings which are believed characteristic of obese persons trying to lose weight [11-13]. The instrument consist of 17 statements about weight-related feelings and emotions which are rated on a 7 point Likert scale ranging from 0 (“not at all”) to 6 (“very much”). The 17 items comprise a scale from 0–100, where higher scores indicate better emotional quality of life (emotional HRQL).

The Weight Related Symptom Measure (WRSM) [10] questionnaire, assesses self-reported presence and distress of 20 obesity-specific symptoms. The number of symptoms ranges from 0–20. A total score ranges from 0 to 120 where higher scores indicate worse symptom distress.

The short form of the Medical Outcome Study (SF-36) [14] is a generic measure of HRQL based on 36 questions measuring functional status and well-being. One question is not scored and measures health change the previous year. The remaining 35 items form eight health domains which can be combined into two summary scales, the physical and mental dimensions. Each scale ranges from 0 to 100 where higher scores indicate better HRQL. We studied only the physical and mental dimensions (physical and mental HRQL) [15,16].

Sense of coherence (SOC) [17] was measured by the orientation to life questionnaire. This questionnaire measures an individual’s capacity to respond to stressful situations. The 13-item questionnaire forms a total score ranging from 13 to 91, where higher scores indicate stronger SOC.

All of the above self-management questionnaires are widely used and have been shown to be valid and reliable in many cultures and populations [10,16,18].

The primary outcome variable in this study was percentage 1-year excess weight loss (EWL). EWL is defined as weight loss in kg exceeding calculated body weight at BMI 25 kg/m^2^. EWL% and weight loss in percent of baseline weight (WL%) were highly correlated in our sample (r = .962), but EWL% had a greater range and variance compared to WL%. Given these considerations, we chose EWL% as dependent variable in the analyses.

In addition to baseline variables, process variables (weight, diary adherence and frequency of GP-visits) were recorded at 12 weeks. These recordings were performed on the first or second day of the second stay. At the first consultation of the second stay at 12 weeks, the nurse recorded whether patients had used the predefined food and activity diary. The options were: not ever, sometimes and daily. In the same consultation GP-visits were recorded: never, monthly, and more often than monthly.

### Statistical methods

All data are presented as mean (SD) or number (%) as appropriate, unless otherwise stated. Chi-square (χ^2^) or Fisher’s exact test was used to analyze categorical data, whilst independent samples t-test was used to analyze continuous data.

Two exploratory linear multivariate regression analyses were performed. To reduce the risk of instability in the regression models (type I and II-errors) due to inclusion of a large number of random variables, 21 theoretical predictors were selected for further analyses. The first linear regression analysis was performed with 1-year EWL as dependent variable, and baseline and process variables as independents. In this analysis 4 variables were significantly associated with the 1 year EWL. The 12 week EWL was highly significant (p < .001). This led to a second linear regression analysis with the same independents and with 12 week EWL as dependent. In this second analysis we found 3 variables significantly or near-significantly associated with the dependent. Examining the Variation of Inflation Factors (VIF) in the models we found no multicollinearity between the selected independent variables.

To explore and test the theoretical relationships between the 7 variables from the exploratory multiple linear analyzes we developed a path model through structural equation modeling. Error terms were fitted to all variables, except age. Full information maximum likelihood estimation was performed in the analyses. The pathways from age and all other variables, from 12 week EWL to 1-year EWL and to 1-year EWL from all other variables, were considered unidirectional, leaving the pathways between the other variables to be tested. The path model was developed by testing different theoretical pathways between the variables until the model fit the data. The model fit was determined by examining the χ^2^, goodness-of-fit index (GFI), and the root mean square error of approximation (RMSEA). The standardized regression coefficients are presented in the diagram on each pathway. We also report squared correlation (r^2^) values to indicate the total variance explained.

Missing values were calculated using multiple imputations (MI). MI is based on a prediction model containing variables theoretically associated with the variables with missing values. The missing values are predicted using existing values from other pre-defined variables in an imputation model. The MI gives more reliable results when the missing values are missing completely at random (MCAR). MI involves three distinct steps. First plausible values for the missing data are filled in M times to generate M complete datasets. Second, the M complete datasets are analyzed using standard statistical methods. Thirdly, the results from the M analyses are combined (pooled). Little's test of missing data showed that the missing data were MCAR (p = 0.173). Body weight at baseline and 1-year together with gender, age, and income were predictor variables in the MI-model. The variables with missing data were both predictor and imputation variables in the MI-model. Through a fully conditional specification, applying linear regression as prediction method for variables at scale level, and two-way interaction for categorical variables, we generated M=5 complete imputed datasets with 10 iterations per dataset. M=5 datasets with 10 iterations were calculated to give a relative efficiency of the imputed data of approximately 95%. Statistical analyses were conducted on each of the 5 complete data sets, and thereafter the multiple analyses results were pooled to achieve single estimates. Observing the fraction of missing information, relative increase variance, and relative efficiency, the imputed data-sets were comparable with the original data-set.

Throughout, we report two-tailed p-values and p<.05 was considered to be statistically significant. The statistical analysis was conducted using SPSS v.19.0 (IBM SPSS Statistics) and Amos 19 (AMOS Development Corp., USA).

## Results

Men had significantly higher body weight, BMI and waist circumference, yearly income and emotional HRQL compared to women. Women had higher percentage of body fat and higher physical HRQL than the men (Table 1).

**Table 1:** Baseline socio-demographic, anthropometric and HRQL characteristics of 199 morbidly obese patients who underwent a 1-year lifestyle intervention programme

**Variable**	**Total n=199**	**Women n=141**	**Men n=58**	**P-value**
Age	45.2 (11.1)	44.4 (11.5)	47.0 (10.1)	.162
Weight (kg)	122.3 (23.4)	114.8 (17.7)	140.5 (25.5)	<.001
BMI (kg/m^2^)	41.9 (6.2)	41.3 (5.6)	43.5 (7.4)	.022
Excess weight (kg)^a^	49.9 (19.6)	45.2 (15.8)	59.5 (23.9)	<.001
Per cent body fat	47.9 (7.1)	50.0 (4.7)	42.7 (9.1)	<.001
Waist circumference (cm)	123.6 (16.0)	119.0 (13.4)	135.7 (15.8)	<.001
Hip circumference (cm)	129.1 (13.7)	130.1 (13.0)	126.5 (15.1)	.103
Married/cohabitant	87 (44)	59 (55)	28 (48)	.867
Employed work	109 (55)	78 (55)	31 (53)	.842
Income (1.000 NOK)	247 (207)	226 (209)	296 (197)	.010
Years of Education				
<9 years	54 (27)	38 (27)	15 (27)	
9-12 years	97 (49)	65 (46)	32 (55)	
>12 years	48 (24)	38 (27)	10 (18)	.323
Age at onset obesity	20.1 (12.1)	20.4 (12.4)	19.4 (11.6)	.592
Maximum weight (kg)	131.1 (25.9)	123.5 (18.9)	149.6 (31.1)	<.001
Age of maximum weight	43.4 (11.4)	42.6 (11.6)	45.1 (10.8)	.084
SOC-total score^b^	60.1 (12.7)	59.9 (13.5)	60.5 (10.6)	.756
Emotional HRQL^c^	39.8 (21.7)	34.6 (27.0)	52.5 (15.4)	<.001
Physical HRQL^d^	38.5 (9.4)	39.7 (8.7)	35.6 (10.5)	.006
Mental HRQL^e^	43.0 (9.4)	43.2 (9.2)	42.4 (10.1)	.581
Number of symptoms^f^	10.1 (4.2)	10.3 (4.0)	9.6 (4.5)	.291
Symptom distress^g^	33.9 (19.0)	35.4 (19.5)	30.3 (17.4)	.107

Data are given as mean (SD) or n (%).

^a^. Weight exceeding BMI=25 kg/m^2^, ^b^. SOC-13, score range from 13–92 (higher score indicate better), ^c^. OWLQOL score range from 0–100 (higher score indicate better), ^d^. SF-36 physical dimension, score range from 0–100 (higher score indicate better), ^e^. SF-36 mental dimension, score range from 0–100 (higher score indicate better), ^f^. WRSM score range from 0–20 (lower score indicate better), ^g^. WRSM score range from 0–102 (lower score indicate better).

Hypertension, joint pain, type 2 diabetes, obstructive sleep apnea, and low back pain were the most commonly diagnosed co-morbidities and did not differ significantly between genders (Table 2).

**Table 2:** Ten most frequently occurring baseline diagnoses (ICD-10 codes) of 199 morbidly obese patients who underwent a 12 month lifestyle intervention programme

**Variable**	**Total n=199**	**Women n=141**	**Men n=58**	**P-value**
Hypertension (I10)	79 (39.7)	51 (36.2)	28 (48.3)	.112
Joint pain (M25.9)	76 (38.2)	50 (35.5)	26 (44.8)	.217
Type 2 diabetes mellitus (E11)	40 (20.1)	26 (18.4)	14 (24.1)	.363
Obstructive sleep apnea (G47.33)	38 (19.1)	23 (16.3)	15 (25.9)	.119
Low back pain (M54.5)	34 (17.1)	27 (19.1)	7 (12.1)	.229
Depression (F32.9)	29 (14.6)	24 (17.0)	5 (8.6)	.102
Elevated fasting glucose (R73.0)	22 (11.1)	14 (9.9)	8 (13.8)	.341
Osteoarthritis (M19)	17 (8.5)	15 (10.6)	2 (3.4)	.099
Gastro-esophageal reflux disease (K21.0)	16 (8.0)	11 (7.8)	5 (8.6)	.848
Anxiety (F41.0)	15 (7.5)	11 (7.8)	4 (6.9)	.827

Data are given as n (%).

At 12 weeks and 1-year the mean (SD) weight loss was 6 (5) kg and 10 (11) kg. By comparison, men lost more weight in kilograms and a higher percentage of fat, whilst women experienced a larger increase in emotional HRQL and lost more cm in hip circumference (Table 3).

**Table 3:** Changes in anthropometric and HRQL characteristics of 199 morbidly obese patients during a 1-year lifestyle intervention programme

**Variable**	**Total n=199**	**Women n=141**	**Men n=58**	**P-value**
Weight (kg)	-10.0 (10.5)	-8.9 (9.9)	-12.6 (11.4)	.022
Weight percent	-8.0 (8.2)	-7.6 (8.7)	-8.7 (7.7)	.396
Excess weight (%)^a^	-20.3 (21.8)	-19.8 (22.9)	-21.4 (18.8)	.652
BMI (kg/m^2^)	-3.4 (3.7)	-3.3 (3.7)	-3.7 (3.8)	.459
Per cent body fat	-3.3 (4.8)	-2.8 (4.4)	-4.6 (5.4)	.026
Waist circumference (cm)	-10.8 (8.8)	-10.2 (8.6)	-12.4 (9.1)	.136
Hip circumference (cm)	-9.3 (8.1)	-10.1 (8.3)	-7.3 (7.3)	.035
SOC-total score^b^	4.3 (11.1)	4.5 (11.0)	3.7 (11.5)	.675
Emotional HRQL^c^	15.9 (21.3)	18.7 (21.4)	9.2 (17.7)	.009
Physical HRQL^d^	5.5 (9.0)	5.7 (8.4)	5.2 (10.2)	.785
Mental HRQL^e^	3.7 (9.4)	4.4 (9.0)	2.1 (10.3)	.196
Symptom distress^f^	-11.6 (15.5)	-12.2 (16.0)	-10.0 (14.1)	.375
Number of symptoms^g^	-2.6 (3.8)	-2.8 (3.7)	-2.2 (4.1)	.375

Data are given as mean (SD).

^a^. Weight exceeding BMI=25 kg/m^2^, ^b^. SOC-13, score range from 13–92 (higher score indicate better), ^c^. OWLQOL score range from 0–100 (higher score indicate better), ^d^. SF-36 physical dimension, score range from 0–100 (higher score indicate better), ^e^. SF-36 mental dimension, score range from 0–100 (higher score indicate better), ^f^. WRSM symptom distress score range from 0–20 (lower score indicate better), ^g^. WRSM number of symptoms score range from 0–120 (lower score indicate better).

There were no significant differences at baseline, 12 weeks or 1 year, in terms of patients having 4 (n = 104) or 5 stays (n = 95) in the intervention period, except in terms of higher income (p = .027) and a somewhat higher number of obesity symptoms (p = .043) at baseline in the group of patients with 4 stays (data not shown).

In the first linear regression analysis we found that age, employment status, mental HRQL, and 12 week EWL were significantly associated with 1-year EWL. The second linear regression analysis showed that type 2 diabetes, frequency of GP-visits, and adherence to the food and activity diary were associated with 12 week EWL (Table 4).

**Table 4:** Linear multiple regression analyses with a. 12 week excess weight loss as dependent, and b. 1-year excess weight loss as dependent

	**a. dependent: 12 week excess weight loss**		**b. dependent: 1-year excess weight loss**	
	**Std. coeff.**	**P-value**	**Std. coeff.**	**P-value**
Baseline variables				
Gender^a^	.062	.467	-.032	.621
Age	.113	.278	.189	.014
Baseline excess weight^b^	-.066	.440	.072	.258
Married/cohabitant^c^	.035	.666	-.017	.775
Employed work^d^	.023	.982	.137	.034
Income	.048	.556	.028	.647
Years of Education	.118	.133	-.095	.132
Age at onset obesity	.018	.934	.021	.824
Baseline SOC-total score^e^	.044	.664	.064	.414
Emotional HRQL^f^	.023	.806	.034	.645
Baseline symptom distress^g^	.033	.796	.042	.641
Physical HRQL^h^	.092	.518	.034	.743
Mental HRQL^i^	-.103	.442	-.227	.033
Hypertension (I10)	-.044	.576	-.055	.341
Joint pain (M25.9)	-.074	.304	.040	.462
Type 2 diabetes mellitus (E11)	-.143	.051	-.035	.519
Obstructive sleep apnea (G47.33)	.006	.984	.021	.699
Low back pain (M54.5)	.075	.311	.055	.320
Process variables (0–3 months)				
Adherence to diary	.152	.059	.017	.789
Frequency of GP-visits	.221	.006	-.010	.927
12 week excess weight loss	-	-	.664	<.001

^a^. females=0, ^b^. Excess Weight (kg. > BMI=25), ^c^. Not married/cohabitant=0, ^d^. Not employed=0, ^e^. Sense of Coherence, score range from 0–92 (higher score indicate better), ^f^. OWLQOL, score range from 0–102 (higher score indicate better), ^g^. WRSM symptom distress score range from 0–120 (lower score indicate better), ^h^. SF-36 physical dimension, score range from 0–100 (higher score indicate better), ^i^. SF-36 mental dimension, score range from 0–100 (higher score indicate better).

Pooled standardized coefficients and p-value are reported.

**Figure 2:**
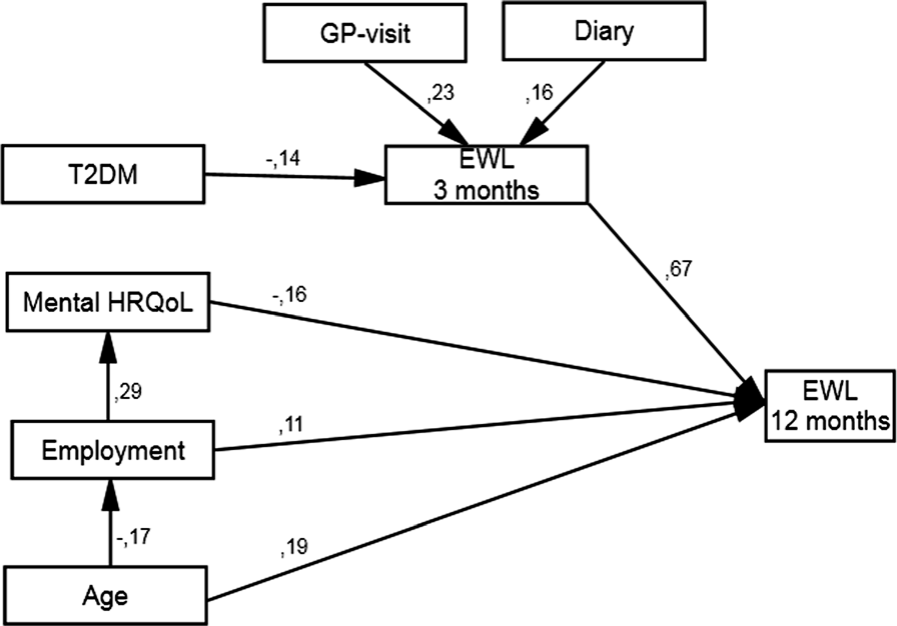
**Model describing paths and strength of associations between predictors of 1-year excess weight loss in 199 morbidly obese patients undergoing intensive lifestyle intervention.** T2DM = Type 2 diabetes mellitus, Mental HRQL = SF-36 mental composite score, GP = general practitioner, EWL = excess weight loss. All values are standardized regression weights (r).

The path-model (Figure 2) shows that work status, 12 week EWL, age, and baseline mental HRQL had direct effects on 1-year EWL. The strongest primary predictor was 12 week EWL (r = .67, p < .001). The frequency of GP-visits, type 2 diabetes, and diary adherence had an indirect effect through an impact on 12 week EWL. Age had a direct effect on 1-year EWL and an indirect effect through employment status. Employment had also a direct effect on 1-year EWL and an indirect effect through mental HRQL. The model had an acceptable fit to the data with χ^2^ = 33.163 (DF = 19, p = .023), CFI = .921 and RMSEA = .061. The squared multiple correlations of the dependent variable indicate that the model explains approximately 50% of the variation (r^2^ = .504) in 1-year EWL.

## Discussion

Our main finding was that weight loss at 12 weeks, age, mental HRQL and employment status had direct effects on weight loss at 1 year. 12 week weight loss had the strongest direct effect.

The impact of initial weight loss on long term weight loss is well described in several studies [19-21]. In a recent American study of 1,685 multi ethnic obese participants, weight loss at 6 months was found to be a consistent predictor of weight loss after 36 months across gender and ethnic groups [22]. The same was reported in a Swedish study of 247 participants undergoing a two-step weight loss program lasting for 8–10 months. The strongest factor for predicting weight loss in the Step II treatment was Step I weight loss. Each 1 kg weight loss in Step I predicted 13% of the variation in Step II weight loss [23]. The randomized multi center Look Ahead-study found that the larger a participant’s weight loss was after the first year, the larger their loss at year 4. The odds of achieving a loss ≥ 10% of baseline weight at year 4 were 9.8 (95% CI: 6.99–13.74) times greater for participants who lost ≥ 10% at year 1 compared to participants who lost < 5% at year 1 and 2.0 (95% CI: 1.41–2.96) times greater for participants who had lost 5.0–9.9% at year 1 compared with those who lost < 5% at year 1 [24].

Our findings support these earlier findings, but also point to certain patient characteristics as significant additional determinants of weight loss. Our analyses show that mental HRQL has a direct effect on 1-year EWL. The mental HRQL scale is based on 13 items measuring vitality, social functioning, emotional role functioning, and mental health, and contains questions like degree of being worn out, being tired, whether emotional problems interfered with work or social activities, and degree of nervousness. Contrary to what might be a common clinical assumption, lower mental HRQL was associated with greater 1-year EWL. This connection is underexplored in lifestyle interventions for the morbidly obese. A systematic review of psychological and psychosocial predictors of weight loss after bariatric surgery included 29 studies with either a retrospective or prospective design [25]. The majority of the studies could not identify psychiatric comorbidity as a negative predictor of weight loss, and some of the studies even found that increased psychological distress, as assessed through higher levels of depression and anxiety, elevated psychiatric scores and low self-esteem before surgery even appeared to be positively associated with weight loss after surgery. A careful interpretation of our findings may be that morbidly obese patients with a lower mental HRQL have a greater motivation to achieve lifestyle changes. A number of studies support this interpretation. A Norwegian qualitative study of obese patients attending a 40-hour patient education course before bariatric surgery revealed through interviews that bodily pain and depression were motivational factors for seeking treatment rather than the size of the body itself [26]. Obesity-associated psychological distress such as low self-esteem, depression and anxiety, and social phobia resulting in social isolation are associated with lower mental HRQL [27].

Only one patient dropped out of the 1 year program. We believe that the group-based focus, the motivational approach, follow up at home and the repeated stays accounted for the low drop-out rate in this study.

In our study employment was a predictor of weight loss. The association between being unemployed and experiencing lower physical and mental health in morbid obesity has been described earlier [28]. It could be argued that employed patients have a more socially challenging and meaningful everyday life. In the same way it could be argued that unemployed patients might experience a lack of inclusion and belonging, being excluded from access to both working and social networks. This notion is supported by studies of cancer patients which have shown positive associations between social networks, support and HRQL [29]. On the other hand, a study of predictors of weight loss after bariatric surgery found that unemployment and work status were not predictors of post-surgical weight loss [25]. Our data suggests that determining employment status may be an important factor when identifying patients in need of extra support during a morbid obesity lifestyle intervention program.

Age was also a found to be a primary predictor of weight loss. Our interpretation, based on our clinical experience, is that older patients are more experienced and goal oriented than younger ones. Greater health concerns among older patients, higher impact of co-morbidities, and HRQL impairments may produce greater motivation for lifestyle change [19].

Age had, in addition, an indirect effect as a mediator on employment status. Older patients are more likely to be unemployed than younger patients. Employment status seemed to affect mental HRQL. Older patients are also more prone to co-morbidities that can negatively affect upon employment. This paints a complex reality; age itself had a direct effect on 1-year EWL and the oldest patients had the highest 1-year EWL. However, being younger seemed to affect positively upon employment status, which in return affected 1-year EWL. The model may imply that younger employed patients have higher 1-year EWL and that employment status mediates the effect of age on EWL. Likewise, employment status affected mental HRQL positively. While higher mental HRQL had a direct negative effect on 1-year EWL, this effect seemed to be mediated by the employment status of the patient.

The positive effect of keeping a food diary on weight loss is well known, and best described in internet based lifestyle programs. In a study of 3,621 subscribers of an internet based weight loss program, participants with high adherence to a food diary were more likely to achieve clinically significant weight loss [30]. An obesity management review found a consistent and significant positive relationship between self-monitoring and weight loss [31]. The positive effect of GP-visits is also well documented. In a New Zealand cluster randomized trial of 750 patients receiving counseling from their GP’s in a 12 month period, mean total energy expenditure and the performance of leisure exercise increased significantly. The proportion of patients undertaking 2.5 hours/week of physical activity was 10% (p = .003) higher in the intervention group compared to the control group [32].

In conclusion, the between-person variances of weight loss following non-surgical weight loss programs vary greatly [7], suggesting such programs to be individualized rather than standardized. In this study of morbidly obese patients following a partly residential weight loss program we found that personal factors like age, mental HRQL and occupational status had a direct effect on 1-year EWL. Notably, neither baseline diagnostic variables, sense of coherence, nor physical conditions or anthropometric characteristics had significant direct effects. Personality characteristics were not assessed and such studies may give additional information on where patients’ focus should lie. However, the best marker of weight loss success after 1 year was 12 week EWL. This is potentially important, because short term weight loss is relatively easily measured, and patients not capable of meeting acceptable short term weight loss goals should be met with increased attention [33]. Maintaining a self-monitoring instrument like a diary, allied with regular visits to a GP for follow up, affect short term weight loss, and we suggest that such tools should be implemented in weight loss programs.

Our study has a number of limitations. The study design was observational, thereby reducing the ability to determine causal effects. All participants were Caucasian, which may reduce generalizability to non-white groups. The selection of participants, morbidly obese patients referred from hospitals, may have been biased. Only a proportion of all morbidly obese seek treatment for their obesity, as such there may be disparities between the study group and the general morbidly obese population.

## Conclusion

Diagnostic, anthropometric, socio-demographic, psychosocial, treatment, and health related quality of life characteristics were examined as possible predictors of excess weight loss after a 1-year partly residential intensive lifestyle program. We found that excess weight loss at 12 weeks, baseline mental HRQL, occupational status, and age had a direct effect on excess weight loss at 1 year.

## Competing interests

Tor-Ivar Karlsen is a PhD-fellow at the Morbid Obesity Centre and works at the University of Agder. He is supported financially through an unrestricted educational grant from Evjeklinikken AS. The other authors declare that there is no conflict of interest that could be perceived as prejudicing the impartiality of the research reported.

## Authors’ contributions

TIK designed the study and collected data from patients. TIK and MS analysed the data and drafted the manuscript. JH revised and helped draft the manuscript. All authors read and approved the final manuscript.
